# Key concepts in artificial intelligence and technologies 4.0 in services

**DOI:** 10.1007/s11628-023-00528-w

**Published:** 2023-02-27

**Authors:** Russell W. Belk, Daniel Belanche, Carlos Flavián

**Affiliations:** 1 Russell W. Belk Schulich School of Business-York University, York University, Toronto, Canada; 2 Department of Marketing Management and Market Research, Faculty of Economy and Business, University of Zaragoza, Zaragoza, Spain; 3 Department of Marketing Management and Market Research, Faculty of Economy and Business, University of Zaragoza, Gran Vía 2, 50005 Zaragoza, Spain

**Keywords:** Artificial intelligence, Technologies 4.0

## Abstract

The emerging Industry 4.0 technologies that are impacting the global economy also represent an extraordinary opportunity to increase customer value in the service sector. Indeed, the ongoing Fourth Industrial Revolution differs from previous technologies in three main ways: (1) technological developments overcomes humans’ capabilities such that humans or even companies are no longer controlling technology; (2) customers embrace life in new technology-made environments, and (3) the boundaries between human and technology become to be blurred. This document explains these novel insights and defines the key AI-related concepts linked to each of these three distinctive aspects of Technologies 4.0 in services.

## Introduction

The emerging Industry 4.0 technologies (also known as Technologies 4.0) represent a great opportunity to increase customer value in the service sector (Lee and Lee 2020). These advanced technologies incorporate disruptive analytical systems and hardware such as: Artificial Intelligence (AI), autonomous robots, virtual and augmented reality (VR/AR), Big Data analytics, cloud computing and the Internet of Things (IoT). The incorporation of Technologies 4.0 in business operations is fast and unstoppable, with AI playing a crucial role in many industries including services. The actual statistics indicate that global corporate investment on AI technologies grew a 38.9% in 2020 and a 37.8% in 2021 (Statista 2022a). AI is being implemented globally disrupting labor markets, with some countries leading this race. In particular, PwC (2022) foresee that the impact of AI on the GDP of the USA and China will be a 14.5% and a 26% boost respectively by 2030. For instance, recent studies suggest that financial services will be totally automatized in seven years, surpassing the level of automation in manufacturing (PwC 2022). However, previous research on Technologies 4.0 has been mostly focused on their impact on manufacturing and supply chain operations (Alcácer and Cruz-Machado 2019), ignoring their tremendous potential to shape current and future service interactions with customers.

Over the history of industry, the introduction of technology has shaken the established status quo of companies’ operations and the whole economy. The first three technological revolutions changed business, labor policies and quality of life. However, the Fourth Industrial Revolution that has already started is moving at an unprecedented rate to alter these standards. The advent of the Fourth Industrial Revolution goes several steps further and differs from previous technologies in three main ways: (1) technological developments are overcoming humans’ capabilities such that humans or even companies are no longer controlling technology, but technology itself starts to set the rules; (2) customers embrace life in new technology-made environments (e.g. social media, virtual worlds, smart devices around personal space), and (3) the boundaries between human and technology become to be blurred (e.g. robots with human skills, humans with integrated technologies).

**Fig. 1:**
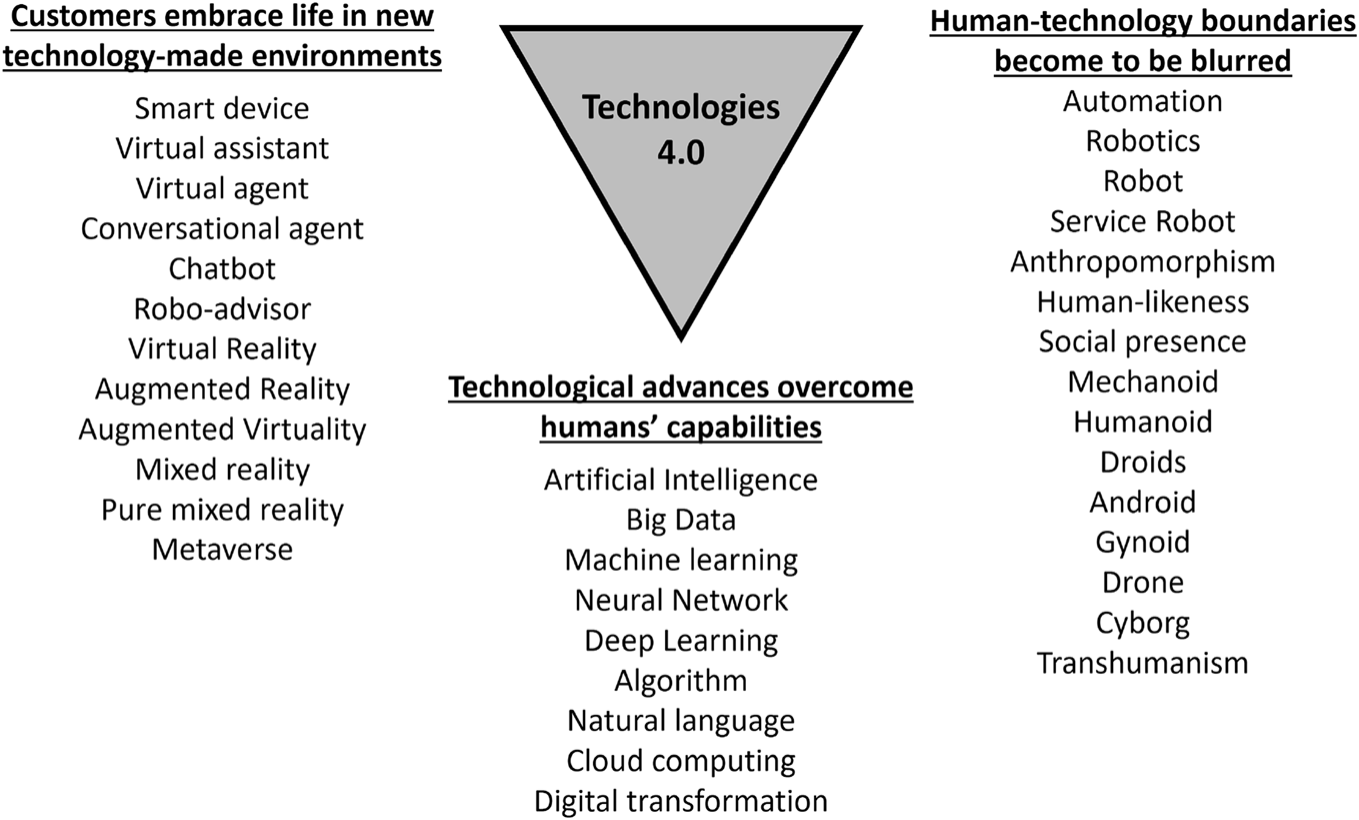
The three distinctive factors of the 4th Industrial Revolution and key concepts

Therefore, our work explains these novel insights, devoting one section to each of the three distinctive aspects of the Fourth Industrial revolution from a service business approach. Given the jigsaw puzzle of technologies and concepts investigated under this research approach, we clarify and define the key concepts related to AI and Technologies 4.0 linked to each section. Figure 1 depicts this framework and the key concepts defined in this work.

## The technology realm based on AI (artificial intelligence) capabilities

AI can be considered an umbrella term integrating many other related concepts such as the technologies enabling AI and the AI capabilities. Table 1 defines AI and other closely related concepts. As a distinctive feature of AI, for the first time the system that embeds abilities/intelligence is technological, not biological. That is, AI shows some aspects of intelligence, meaning that at least to some extent AI is able of adapting to different contexts and of learning from experience. The concepts of machine learning and, one step further, deep learning, refers to this new ability of technology. Thus, systems integrating AI are totally different from automation implemented in factories thirty years ago that were just based on task repetition. Actually, AI does repeat tasks but this process helps the system to perform the task each time in a better way, imitating the human learning and adaptation process. Mathematical algorithms apply rules to improve solutions to a task, and current advances in neural networks suggest that the association of elements carried out by technological systems (operating as brains apparently do) is a good strategy for learning.

**Table 1:** Definitions of key concepts on AI and assimilated technologies

Artificial intelligence	Capacity to combine perception, reasoning, learning and actuation by an autonomous non-biological system replicating or exceeding human abilities, or the system exhibiting that capacity
Big data	Very large sets of information often collected by digital technologies and that require sophisticated analytical tools to be processed
Machine learning	Process by which AI generates its own rules to carry out a task adapted to data feed without human intervention
Neural network	Artificial system that imitates the way the brain presumably works to associate elements and learn autonomously without previously scripted rules
Deep learning	Advanced level of machine learning that aims to replicate the understanding of a phenomenon by a human brain
Algorithm	A set of rules or mathematical instructions that process and analyze information (input) to find a solution (outcome) for a given problem
Natural language	Computer language that processes or replicates the current way people communicate with others rather than using artificial languages that are difficult to interpret by humans
Cloud computing	Service that stores data and runs software on a large-scale digital server connected to the internet rather than on a computer
Digital transformation	Organizational process based on the digitalization of products, services or other operations that relies on Technologies 4.0, the Internet and related technological systems (e.g., mobile apps) to increase value and customer satisfaction

Due to its analytical capabilities (Huang and Rust 2021), AI makes decisions by relying on large amounts of data (Big Data), data that humans are unable to handle or analyse. To improve the performance and accessibility of interconnected AI to this information (e.g. digital records), cloud computing is often employed as the online system to store these huge amounts of data. An example, in the successful business of Amazon: AI makes decisions related to shipments’ logistics in real time thanks to machine learning without human intervention or comprehension. As a consequence of these abilities’ improvements, AI not only improves labour productivity, saves time and improves quality, but also perform well in new abilities such as service personalization (PwC 2022). For instance, AI capabilities exceed human skills in many mechanical and analytical tasks such as robo-advisory services analyzing investment parameters (Flavián et al. 2022). In this Special Issue, the article by Chin et al. (2023) analyzes previous studies about the digital transformation carried out by service business that involves the Internet and recent Technologies 4.0. From a complementary approach and focused on environmental issues, Pandya and Kumar (2023), identify the 4.0 Technologies that will have the greatest impact on sustainability.

## Living in a technology-led environment

Society is gradually moving from natural environments to digital and virtual ones. For instance, global social media penetration rate is 58.4%, with people in their forties spending almost 60 min per day on social media (Statista 2022b). Social media users spend their time on these platforms to stay connected with family and friends, filling spare time, looking for inspiration and reading news stories (Statista 2022b). Based on advertising and commercial motivations, social media algorithms decide what to show in user’s screen each morning and what to be hidden.

There are many other examples corroborating the disruptive irruption of Technologies 4.0 in people’s lives, Table 2 defines some of the key concepts in this area. Linking the physical to the digital worlds, the IoT (Internet of Things) is an emerging and evolving phenomenon in the 5G digital era. Like smart sensors employed to improve customers’ health (Gelbrich et al. 2021), all kind of devices connected to the internet (e.g., household appliances, devices for security monitoring) entail a great opportunity to increase customer value by expanding current services with a technological focus. Virtual assistants providing advice and helping in managing daily activities (e.g. playing music) are one of the clearest examples of how to integrate technology in people’s homes.

**Table 2:** Definitions of key concepts on virtual and AI-related environments

Internet of Things (IoT)	System created by a network of devices connected to each other that embeds software and sensors and exchanges data through the internet
Smart device	Technological object that shows intelligence or works independently and that is connected to the internet to enhance its interaction with the user
Virtual assistant	Smart device or software connected to the internet that embeds AI to assist an individual user in his/her routine tasks or to perform actions according to user’s orders by means of voice or text commands
Virtual agent	Artificial system that interacts with customers to perform tasks in a manner similar to a human agent including information provision, sales or customer service
Conversational agent	Software that by means of algorithms automates conversations with users including answering questions and that simulates human communication
Chatbot	Type of conversational agent, often text-based, that is designed with the purpose of providing users with a specific service
Robo-advisor	Technological software acting as an advisory agent that is able to automate or assist customers in managing their financial investments
Virtual reality	Environment created by computer where users can interact and simulate actions having a sensory immersive experience in real time
Augmented Reality	Scenario resulting from the overlapping of computer-generated images superimposed on a view of the real world
Augmented Virtuality	Scenario resulting from the superimposition of images from the real world on a computer-generated scenario
Mixed reality	Merging of the real and virtual worlds where both kinds of objects or worlds co-exist in the same experience for the user
Pure mixed reality	Scenery resulting from the perfectly overlapping of realistic computer-generated images and real-world images with seamless integration
Metaverse	Shared collective virtual space, in which users can interact with each other through avatars or with virtual objects and environments in real time. It is created through the convergence of virtually enhanced physical reality and physically persistent virtual space

Technologies 4.0 has also enhanced the dynamic capabilities of organizations to rapidly respond to the evolving customer needs and preferences (Lee and Lee 2020). They are challenging traditional front-line service encounters, especially in a post-Covid-19 era where “contactless” services make it easy to avoid face-to-face contact between employees and consumers (Lee and Lee 2020). Conversational and virtual agents, commercial chatbots and robo-advisors guiding financial investments are examples of this shift to more user-oriented technology-led service encounters. In this Special Issue Camilleri and Troise (2023) investigate how chatbots mimic human customer services’ abilities, and Akdim and Casaló (2023) analyze the customers’ value and engagement in relation to these voice assistants.

Going one step further, virtual, augmented and mixed realities (VR/AR/MR) rely on highly immersive technologies to generate unique sensory, affective, cognitive and behavioral customer experiences that can significantly affect their satisfaction. VR/AR is being successfully implemented in sectors such as education and healthcare and presents particular advantages to be developed in the tourism and entertainment industries (Flavián et al. 2019). These technologies are also essential for the development of metaverse. These immersive experiences move the user to a new setting with various virtual components and great realism, achieving increasingly complete sensory experiences, which may enhance previous or later experiences with some traditional services and may even replace them. As a recent example, the 2022 AIRSI conference supporting this Special Issue was partially celebrated in the metaverse. The article by Lehtonen et al. (2023) in this Special Issue shows how mobile games influence players’ identity extensions depending on company’s monetization strategies.

## Human and technology, closer than friends

Thanks to AI, the advance of automation achieved in the last decades should be rapidly expanding to all sectors including service industries (PwC 2022). However, contrary to elementary robotics focused exclusively on the mechanization of human tasks, novel robots incorporate many human features and skills. Table 3 describes key concepts related to service robots and other humanized technologies. Anthropomorphism and the level of human-likeness are often considered the most crucial factors affecting customer acceptance of service robots. Mechanoids, humanoids, droids and gynoids are different names assigned to robots depending on their physical appearance. In this Special Issue the articles by Becker et al. (2023) and Molinillo et al. (2023) study anthropomorphism and other crucial factors increasing customers’ acceptance of service robots in restaurants; the Ivanov et al. (2023) assess tourists’ options regarding the introduction of robots in the tourism and hospitality sector.

**Table 3:** Key definitions on robots and other humanized technologies

Automation	Machines or artificial systems that can operate autonomously without human intervention
Robotics	The science of creating and employing robots
Robot	Machine that is capable to perform tasks autonomously and that often physically a replaces humans
Service Robot	Machine or interface that may interact with customers to perform frontline or service operations
Anthropomorphism	Human’s attribution of human features or traits in a non-human entity (e.g. name, face, emotions, will)
Human-likeness	The degree of human physical appearance of a non-human entity
Social presence	Technology capacity to truly engage humans socially such as the sense of being with another or to establish a social relationship
Mechanoid	Robot with a mechanical-like physical appearance
Humanoid	Robot with some human features (e.g. arms, legs, head) in its physical appearance
Droids	Robot with a realistic human like physical appearance
Android	Male droid. Sometimes used as a synonym of droid
Gynoid	Female droid
Drone	Unmanned aerial vehicle
Cyborg	Human who integrates some technology in his/her body to perform some of his/her physiological functions
Transhumanism	A belief that science and technology should help humans to transcend their mental and physical limitations beyond the current limits

Another issue related to the humanization of technology and robots in particular refers to their social presence, that is, their capability to engage customers socially and to establish relationship with them. This innovative skill represents a wide avenue of opportunity for marketing practitioners and scholars, since the human touch employed in relationship-oriented tasks no longer needs to rely on humans (Gelbrich et al. 2021). A particular example of this human–robot relationship are sex robots a thought-provoking phenomenon that is expanding nowadays (Belk 2022). A challenge in the development of service robots is the development of empathy and emotions in these technological entities. Human emotions are different from any kind of artificial emotion (Huang and Rust 2021; Belk 2022), suggesting that AI does not really feel or empathize but is just able to identify human emotion and to react accordingly, thus, creating a kind of artificial empathy or emotional reactions but without actual feeling or comprehension. From a labour approach, companies are also starting to work with employee-robot hybrid teams (Wirtz et al. 2022). In this Special Issue, Loureiro et al. (2022) reveal the stress and happiness of employees working with AI agents.

Finally, the gap between human and robot/technology is also becoming smaller if considered from the human side. Exoskeletons allowing workers to lift heavy loads and AI typing tools correcting messages in real time are examples of technological advances helping people in their physical and non-physical daily tasks. When a human integrates this technology in their body, he or she is called a cyborg. In the health sector, many patients who have lost some of their extremities or capabilities due serious harms were able to recover part of their capabilities thanks to technology (implants). Transhumanism advocates for a better world based on freeing humans from their limits thanks to technology. However, as far as technology is not ruled by biological laws, the integration of technology in the human body beyond medical purposes (e.g. to hear or see better than anyone’s before) remains as a controversial issue.

## Conclusions

AI and other Technologies in Industry 4.0 differ from technologies in previous Industrial Revolutions because (1) they have better abilities than humans which based on technology assuming the control of certain business operations, (2) people are gradually living in virtual or technology-led worlds representing a marketing opportunity, and (3) the boundaries between human and technology (e.g. robots) are blurred suggesting closer collaboration, coopetition and integration. By means of this work, we encourage researchers to explore these distinctive features and the challenges that they represent for the services marketing field. Technologies 4.0 should no longer be treated just as another technology implemented by companies because AI, VR and service robots are actually reshaping business operations, employees’ roles and customers’ places in service interactions.

To help scholars contribute to the understanding of these technologies from a service perspective, our work clarifies the key concepts related to Technologies 4.0. This list of terms is not exhaustive, and many other aspects of these new technologies (e.g. authenticity, warmth, creativity, intuition) also deserve further research attention.

Finally, despite the clear advantages of Technologies 4.0 in services, their implementation may also raise important concerns among customers, employees and managers of services industries which is likely to lead to controversial issues in society and public policy (Belanche et al. 2020; Belk 2021). These problems will affect various ethical and privacy issues arising from the possible misuse of these new technologies, or because of dehumanizing impacts on relationships between people through the increasing intermediation of these technologies (Belanche et al. 2021; Belk 2020). The growing incorporation of all these technologies requires a deep reflection on the role that technology should play in our society (Belk 2013) and how far we are willing to go.

## Data Availability

In this theoretical paper it is not appropriate to make any statement regarding the availability of data.
